# 2-Amino-5-bromo­pyridine–4-hy­droxy­benzoic acid (1/1)

**DOI:** 10.1107/S1600536810025924

**Published:** 2010-07-07

**Authors:** Ching Kheng Quah, Madhukar Hemamalini, Hoong-Kun Fun

**Affiliations:** a X-ray Crystallography Unit, School of Physics, Universiti Sains Malaysia, 11800 USM, Penang, Malaysia

## Abstract

The title 1:1 adduct, C_5_H_5_BrN_2_·C_7_H_6_O_3_, contains two mol­ecules of each species in the asymmetric unit, with similar geometries. In the crystal, mol­ecules are linked to form extended chains along [100] by N—H⋯O, O—H⋯O, O—H⋯N and C—H⋯O hydrogen bonds. Adjacent chains are crosslinked *via* further N—H⋯O inter­actions into sheets lying parallel to (001). The crystal studied was an inversion twin with a 0.54 (2):0.46 (2) domain ratio.

## Related literature

For substituted pyridines, see: Pozharski *et al.* (1997 ▶); Katritzky *et al.* (1996 ▶). For details of hydrogen bonding, see: Scheiner (1997 ▶); Jeffrey & Saenger (1991 ▶); Jeffrey (1997 ▶). For 4-hy­droxy­benzoic acid, see: Vishweshwar *et al.* (2003 ▶). For related structures, see: Hemamalini & Fun (2010*a* ▶,*b* ▶,*c* ▶); Quah *et al.* (2008*a* ▶,*b* ▶, 2010 ▶). For reference bond lengths, see: Allen *et al.* (1987 ▶). For the stability of the temperature controller used for the data collection, see: Cosier & Glazer (1986 ▶).
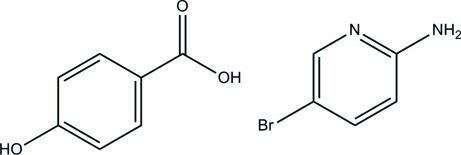


## Experimental

### 

#### Crystal data

C_5_H_5_BrN_2_·C_7_H_6_O_3_
*M* *_r_* = 311.14
Orthorhombic, 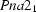

*a* = 21.370 (12) Å
*b* = 3.990 (2) Å
*c* = 28.939 (15) Å
*V* = 2467 (2) Å^3^
*Z* = 8
Mo *K*α radiation
μ = 3.33 mm^−1^
*T* = 100 K
0.29 × 0.12 × 0.09 mm

#### Data collection

Bruker SMART APEXII DUO CCD diffractometer
Absorption correction: multi-scan (*SADABS*; Bruker, 2009 ▶) *T*_min_ = 0.449, *T*_max_ = 0.763
6994 measured reflections
3770 independent reflections
2994 reflections with *I* > 2σ(*I*)
*R*_int_ = 0.051

#### Refinement

*R*[*F* ^2^ > 2σ(*F* ^2^)] = 0.057
*wR*(*F* ^2^) = 0.148
*S* = 1.10
3770 reflections
296 parameters
1 restraint
H-atom parameters constrained
Δρ_max_ = 0.72 e Å^−3^
Δρ_min_ = −0.99 e Å^−3^
Absolute structure: Flack (1983 ▶), 1554 Friedel pairs
Flack parameter: 0.54 (2)

### 

Data collection: *APEX2* (Bruker, 2009 ▶); cell refinement: *SAINT* (Bruker, 2009 ▶); data reduction: *SAINT* ; program(s) used to solve structure: *SHELXTL* (Sheldrick, 2008 ▶); program(s) used to refine structure: *SHELXTL*; molecular graphics: *SHELXTL*; software used to prepare material for publication: *SHELXTL* and *PLATON* (Spek, 2009 ▶).

## Supplementary Material

Crystal structure: contains datablocks global, I. DOI: 10.1107/S1600536810025924/hb5539sup1.cif

Structure factors: contains datablocks I. DOI: 10.1107/S1600536810025924/hb5539Isup2.hkl

Additional supplementary materials:  crystallographic information; 3D view; checkCIF report

## Figures and Tables

**Table 1:** Hydrogen-bond geometry (Å, °)

*D*—H⋯*A*	*D*—H	H⋯*A*	*D*⋯*A*	*D*—H⋯*A*
N2*A*—H2*AA*⋯O3*A*^i^	0.86	2.19	2.996 (11)	155
N2*A*—H2*AB*⋯O1*A*^ii^	0.86	2.13	2.969 (11)	166
N2*B*—H2*BA*⋯O3*B*^iii^	0.86	2.19	3.020 (11)	163
O1*A*—H1*AB*⋯O3*B*^iv^	0.82	1.87	2.688 (8)	175
O2*A*—H2*AC*⋯N1*A*^v^	0.82	1.80	2.605 (10)	168
O1*B*—H1*BB*⋯O3*A*^vi^	0.82	1.94	2.762 (8)	177
O2*B*—H2*BC*⋯N1*B*^vii^	0.82	1.85	2.663 (11)	170
C6*B*—H6*B*⋯O1*A*^viii^	0.93	2.52	3.416 (11)	161
C7*B*—H7*B*⋯O3*A*^vi^	0.93	2.58	3.262 (12)	131
C9*A*—H9*A*⋯O3*B*^iv^	0.93	2.48	3.182 (11)	132
C10*A*—H10*A*⋯O1*B*^ix^	0.93	2.53	3.416 (11)	158

Symmetry codes: (i) 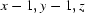; (ii) 
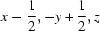
; (iii) 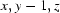; (iv) 
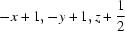
; (v) 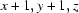; (vi) 
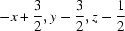
; (vii) 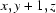; (viii) 
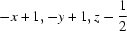
; (ix) 
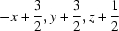
.

**Fig. 1.**
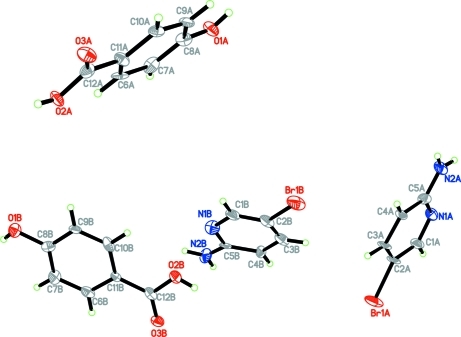
The molecular structure of (I), showing 50% probability displacement ellipsoids for non-H atoms.

**Fig. 2.**
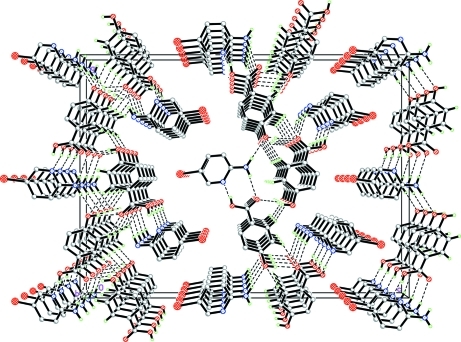
The crystal structure of (I) viewed along the b axis. H atoms not involved in intermolecular hydrogen bond interactions (dashed lines) have been omitted for clarity.

## supplementary crystallographic information

### Comment

Pyridine and its derivatives play an important role in heterocyclic chemistry (Pozharski *et al.*, 1997; Katritzky *et al.*, 1996). They are often involved in hydrogen-bond interactions (Jeffrey & Saenger, 1991; Jeffrey, 1997; Scheiner, 1997). 4-Hydroxybenzoic acid is a good hydrogen-bond donor and can form co-crystals with other organic molecules (Vishweshwar *et al.*, 2003). We have recently reported the crystal structures of 2-amino-5-bromopyridine-benzoic acid (Hemamalini & Fun, 2010*a*), 2-amino-5-bromopyridinium 3-aminobenzoate (Hemamalini & Fun, 2010*b*) and 2-amino-5-bromopyridinium hydrogen succinate (Hemamalini & Fun, 2010*c*) from our laboratory. In continuation of our studies of pyridinium derivatives, the crystal structure determination of the title compound has been undertaken.

The asymmetric unit of the title compound consists of two crystallographically independent 2-amino-5-bromopyridine molecules (*A* and *B*) and two 4-hydroxybenzoic acid (*A* and *B*) with comparable geometries. The bond lengths (Allen *et al.*, 1987) and angles in the title compound (Fig. 1) are within normal ranges and comparable with the related structures (Quah *et al.*, 2010, 2008*a*, b). Each 2-amino-5-bromopyridine molecule is approximately planar, with a maximum deviation of 0.020 (8) Å for atom C4A in molecule *A* and 0.021 (8) Å for atom C1B in molecule *B*. In molecule *A*, the 2-amino-5-bromopyridine molecule is inclined at dihedral angle of 28.8 (3) and 55.7 (3)° with the C6A—C11A and C6B—C11B phenyl rings, respectively. The correspondence angles for molecule *B* are 45.6 (3) and 27.2 (3)°.

In the crystal packing, the molecules are linked to form extended chains along [100] by intermolecular N2A–H2AA···O3A, N2B–H2BA···O3B, O–H···O, O–H···N and C–H···O hydrogen bonds (Table 1). The adjacent chains are cross-linked *via* N2A–H2AB···O1A interactions into two-dimensional networks (Fig. 2) parallel to the (001).

### Experimental

A hot methanol solution (20 ml) of 2-amino-5-bromopyridine (43 mg, Aldrich) and 4-hydroxybenzoic acid (34 mg, Merck) were mixed and warmed over a heating hotplate magnetic stirrer for a few minutes. The resulting solution was allowed to cool slowly to room temperature and brown needles of (I) appeared after a few days.

### Refinement

All H atoms were positioned geometrically and refined using a riding model with O—H = 0.82 Å, N—H = 0.86 Å, C—H = 0.93 Å; and *U*_iso_(H) = 1.5 *U*_eq_(O), 1.2 *U*_eq_(N) and 1.2 or 1.5 *U*_eq_(C). The highest residual electron density peak is located at 1.06 Å from BR1B and the deepest hole is located at 0.90 Å from BR1B. The same U^ij^ parameters were used for atom pairs C1A/C1B, C2A/C3A, C2B/C3B, C8A/C8B and C9A/C9B. The reported Flack parameter was obtained by TWIN/BASF procedure in *SHELXL* (Sheldrick, 2008).

### Crystal data

**Table:**

C_5_H_5_BrN_2_·C_7_H_6_O_3_	*F*(000) = 1248
*M**_r_* = 311.14	*D*_x_ = 1.675 Mg m^−^^3^
Orthorhombic, *P**n**a*2_1_	Mo *K*α radiation, λ = 0.71073 Å
Hall symbol: P 2c -2n	Cell parameters from 1665 reflections
*a* = 21.370 (12) Å	θ = 2.4–25.0°
*b* = 3.990 (2) Å	µ = 3.33 mm^−^^1^
*c* = 28.939 (15) Å	*T* = 100 K
*V* = 2467 (2) Å^3^	Needle, brown
*Z* = 8	0.29 × 0.12 × 0.09 mm

### Data collection

**Table:**

Bruker SMART APEXII DUO CCD diffractometer	3770 independent reflections
Radiation source: fine-focus sealed tube	2994 reflections with *I* > 2σ(*I*)
graphite	*R*_int_ = 0.051
φ and ω scans	θ_max_ = 25.0°, θ_min_ = 1.9°
Absorption correction: multi-scan (*SADABS*; Bruker, 2009)	*h* = −25→19
*T*_min_ = 0.449, *T*_max_ = 0.763	*k* = −4→4
6994 measured reflections	*l* = −30→34

### Refinement

**Table:**

Refinement on *F*^2^	Secondary atom site location: difference Fourier map
Least-squares matrix: full	Hydrogen site location: inferred from neighbouring sites
*R*[*F*^2^ > 2σ(*F*^2^)] = 0.057	H-atom parameters constrained
*wR*(*F*^2^) = 0.148	*w* = 1/[σ^2^(*F*_o_^2^) + (0.0538*P*)^2^ + 9.0399*P*] where *P* = (*F*_o_^2^ + 2*F*_c_^2^)/3
*S* = 1.10	(Δ/σ)_max_ < 0.001
3770 reflections	Δρ_max_ = 0.72 e Å^−^^3^
296 parameters	Δρ_min_ = −0.99 e Å^−^^3^
1 restraint	Absolute structure: Flack (1983), 1554 Friedel pairs
Primary atom site location: structure-invariant direct methods	Flack parameter: 0.54 (2)

### Special details

**Table:**

Experimental. The crystal was placed in the cold stream of an Oxford Cyrosystems Cobra open-flow nitrogen cryostat (Cosier & Glazer, 1986) operating at 100.0 (1) K.
Geometry. All e.s.d.'s (except the e.s.d. in the dihedral angle between two l.s. planes) are estimated using the full covariance matrix. The cell e.s.d.'s are taken into account individually in the estimation of e.s.d.'s in distances, angles and torsion angles; correlations between e.s.d.'s in cell parameters are only used when they are defined by crystal symmetry. An approximate (isotropic) treatment of cell e.s.d.'s is used for estimating e.s.d.'s involving l.s. planes.
Refinement. Refinement of *F*^2^ against ALL reflections. The weighted *R*-factor *wR* and goodness of fit *S* are based on *F*^2^, conventional *R*-factors *R* are based on *F*, with *F* set to zero for negative *F*^2^. The threshold expression of *F*^2^ > σ(*F*^2^) is used only for calculating *R*-factors(gt) *etc*. and is not relevant to the choice of reflections for refinement. *R*-factors based on *F*^2^ are statistically about twice as large as those based on *F*, and *R*- factors based on ALL data will be even larger.

### Fractional atomic coordinates and isotropic or equivalent isotropic displacement parameters (Å^2^)

**Table:**

	*x*	*y*	*z*	*U*_iso_*/*U*_eq_	
Br1A	0.00906 (6)	0.7888 (2)	0.30916 (3)	0.0442 (3)	
N1A	−0.0212 (3)	0.348 (2)	0.4357 (3)	0.0213 (18)	
N2A	0.0361 (4)	0.2594 (19)	0.5015 (3)	0.0281 (18)	
H2AA	0.0040	0.1584	0.5127	0.034*	
H2AB	0.0697	0.2776	0.5177	0.034*	
C1A	−0.0273 (5)	0.469 (2)	0.3934 (3)	0.0262 (14)	
H1AA	−0.0657	0.4460	0.3787	0.031*	
C2A	0.0212 (5)	0.628 (2)	0.3699 (3)	0.0265 (15)	
C3A	0.0794 (5)	0.672 (2)	0.3928 (3)	0.0265 (15)	
H3AA	0.1132	0.7740	0.3782	0.032*	
C4A	0.0836 (4)	0.556 (2)	0.4369 (3)	0.021 (2)	
H4AA	0.1206	0.5890	0.4532	0.026*	
C5A	0.0338 (4)	0.389 (2)	0.4585 (3)	0.020 (2)	
Br1B	0.25717 (6)	0.3515 (2)	0.37657 (4)	0.0456 (3)	
N1B	0.2861 (4)	−0.067 (2)	0.2491 (3)	0.0265 (19)	
N2B	0.2263 (4)	−0.178 (2)	0.1857 (3)	0.030 (2)	
H2BA	0.2591	−0.2604	0.1728	0.036*	
H2BB	0.1912	−0.1760	0.1711	0.036*	
C1B	0.2925 (5)	0.049 (2)	0.2930 (3)	0.0262 (14)	
H1BA	0.3312	0.0346	0.3075	0.031*	
C2B	0.2442 (5)	0.183 (2)	0.3155 (4)	0.0276 (16)	
C3B	0.1851 (5)	0.196 (2)	0.2964 (3)	0.0276 (16)	
H3BA	0.1514	0.2832	0.3129	0.033*	
C4B	0.1773 (4)	0.076 (2)	0.2513 (3)	0.022 (2)	
H4BA	0.1383	0.0822	0.2370	0.027*	
C5B	0.2297 (4)	−0.056 (2)	0.2280 (3)	0.021 (2)	
O1A	0.6515 (3)	0.3077 (15)	0.5579 (2)	0.0242 (15)	
H1AB	0.6471	0.3109	0.5860	0.036*	
O2A	0.8817 (3)	1.0048 (16)	0.4626 (2)	0.0225 (14)	
H2AC	0.9144	1.1065	0.4579	0.034*	
O3A	0.9066 (3)	1.1204 (15)	0.5362 (2)	0.0234 (14)	
C6A	0.7754 (4)	0.662 (2)	0.4870 (3)	0.018 (2)	
H6A	0.7868	0.6712	0.4561	0.022*	
C7A	0.7202 (4)	0.498 (2)	0.5000 (3)	0.023 (2)	
H7A	0.6947	0.3996	0.4777	0.027*	
C8A	0.7043 (4)	0.483 (2)	0.5461 (3)	0.0195 (13)	
C9A	0.7426 (4)	0.634 (2)	0.5785 (3)	0.0179 (14)	
H9A	0.7311	0.6283	0.6094	0.022*	
C10A	0.7957 (4)	0.789 (2)	0.5664 (3)	0.017 (2)	
H10A	0.8212	0.8811	0.5892	0.020*	
C11A	0.8133 (4)	0.813 (2)	0.5202 (3)	0.0180 (19)	
C12A	0.8711 (4)	0.990 (2)	0.5070 (3)	0.020 (2)	
O1B	0.6068 (3)	−0.1993 (15)	0.1278 (2)	0.0235 (14)	
H1BB	0.6039	−0.2558	0.1006	0.035*	
O2B	0.3859 (3)	0.5786 (15)	0.2236 (2)	0.0235 (14)	
H2BC	0.3541	0.6888	0.2282	0.035*	
O3B	0.3558 (3)	0.6584 (16)	0.1505 (2)	0.0215 (14)	
C6B	0.4619 (4)	0.286 (2)	0.1194 (3)	0.023 (2)	
H6B	0.4350	0.3680	0.0968	0.027*	
C7B	0.5150 (4)	0.105 (2)	0.1061 (4)	0.023 (2)	
H7B	0.5234	0.0653	0.0750	0.028*	
C8B	0.5547 (4)	−0.0131 (19)	0.1401 (3)	0.0195 (13)	
C9B	0.5417 (4)	0.031 (2)	0.1864 (3)	0.0179 (14)	
H8B	0.5680	−0.0607	0.2086	0.022*	
C10B	0.4906 (4)	0.210 (2)	0.1995 (3)	0.022 (2)	
H10B	0.4828	0.2442	0.2307	0.026*	
C11B	0.4487 (4)	0.345 (2)	0.1661 (3)	0.0134 (18)	
C12B	0.3937 (4)	0.539 (2)	0.1789 (3)	0.017 (2)	

### Atomic displacement parameters (Å^2^)

**Table:**

	*U*^11^	*U*^22^	*U*^33^	*U*^12^	*U*^13^	*U*^23^
Br1A	0.0924 (9)	0.0243 (4)	0.0159 (5)	−0.0036 (5)	0.0007 (7)	0.0058 (6)
N1A	0.023 (4)	0.025 (4)	0.016 (4)	−0.004 (3)	−0.001 (3)	0.001 (4)
N2A	0.040 (5)	0.029 (4)	0.016 (4)	−0.003 (4)	−0.001 (4)	−0.003 (3)
C1A	0.037 (4)	0.029 (3)	0.013 (3)	0.003 (3)	−0.002 (3)	0.002 (3)
C2A	0.051 (4)	0.014 (3)	0.014 (4)	0.002 (3)	0.008 (3)	0.007 (3)
C3A	0.051 (4)	0.014 (3)	0.014 (4)	0.002 (3)	0.008 (3)	0.007 (3)
C4A	0.030 (5)	0.013 (4)	0.021 (5)	0.000 (4)	0.009 (4)	0.003 (4)
C5A	0.027 (6)	0.012 (4)	0.020 (5)	−0.003 (4)	−0.002 (4)	0.000 (4)
Br1B	0.0919 (10)	0.0266 (5)	0.0183 (5)	0.0062 (5)	0.0027 (6)	−0.0037 (5)
N1B	0.022 (5)	0.028 (4)	0.030 (5)	−0.003 (4)	−0.006 (4)	0.000 (4)
N2B	0.010 (4)	0.040 (5)	0.041 (6)	0.001 (4)	0.005 (3)	0.003 (4)
C1B	0.037 (4)	0.029 (3)	0.013 (3)	0.003 (3)	−0.002 (3)	0.002 (3)
C2B	0.052 (4)	0.012 (3)	0.018 (4)	−0.005 (3)	0.008 (3)	0.000 (3)
C3B	0.052 (4)	0.012 (3)	0.018 (4)	−0.005 (3)	0.008 (3)	0.000 (3)
C4B	0.032 (6)	0.013 (4)	0.023 (5)	0.005 (4)	0.007 (4)	−0.004 (4)
C5B	0.029 (5)	0.013 (4)	0.020 (5)	0.002 (4)	0.001 (4)	0.013 (4)
O1A	0.026 (3)	0.029 (3)	0.018 (4)	−0.003 (3)	0.001 (3)	0.001 (3)
O2A	0.018 (3)	0.036 (4)	0.014 (4)	−0.005 (3)	0.002 (3)	0.003 (3)
O3A	0.031 (4)	0.025 (3)	0.014 (3)	−0.006 (3)	−0.003 (3)	−0.001 (3)
C6A	0.029 (5)	0.012 (4)	0.014 (5)	0.004 (4)	−0.007 (4)	0.003 (4)
C7A	0.020 (5)	0.020 (5)	0.029 (6)	0.000 (4)	−0.008 (4)	−0.006 (4)
C8A	0.024 (3)	0.007 (3)	0.027 (3)	−0.005 (3)	−0.002 (3)	0.000 (3)
C9A	0.021 (4)	0.020 (3)	0.013 (3)	0.000 (3)	−0.003 (3)	0.007 (3)
C10A	0.027 (5)	0.007 (4)	0.017 (5)	−0.001 (4)	−0.003 (4)	0.001 (3)
C11A	0.024 (5)	0.017 (4)	0.013 (5)	−0.004 (4)	0.000 (4)	−0.003 (4)
C12A	0.024 (5)	0.023 (4)	0.013 (5)	0.010 (4)	−0.001 (4)	−0.004 (4)
O1B	0.028 (4)	0.025 (3)	0.018 (4)	0.000 (3)	−0.001 (3)	−0.006 (3)
O2B	0.024 (3)	0.033 (3)	0.014 (4)	0.005 (3)	0.003 (3)	0.003 (3)
O3B	0.021 (3)	0.027 (3)	0.017 (3)	−0.002 (3)	−0.003 (3)	0.005 (3)
C6B	0.029 (5)	0.019 (5)	0.020 (5)	−0.008 (4)	0.002 (4)	0.004 (4)
C7B	0.028 (5)	0.020 (5)	0.021 (5)	0.001 (4)	−0.001 (4)	−0.012 (4)
C8B	0.024 (3)	0.007 (3)	0.027 (3)	−0.005 (3)	−0.002 (3)	0.000 (3)
C9B	0.021 (4)	0.020 (3)	0.013 (3)	0.000 (3)	−0.003 (3)	0.007 (3)
C10B	0.027 (5)	0.029 (5)	0.009 (5)	−0.007 (4)	0.002 (4)	−0.004 (4)
C11B	0.020 (5)	0.011 (4)	0.009 (4)	−0.004 (4)	−0.002 (3)	−0.001 (3)
C12B	0.019 (5)	0.019 (4)	0.014 (5)	−0.011 (4)	0.001 (4)	−0.004 (4)

### Geometric parameters (Å, °)

**Table:**

Br1A—C2A	1.889 (10)	O2A—H2AC	0.8200
N1A—C1A	1.322 (11)	O3A—C12A	1.249 (10)
N1A—C5A	1.359 (11)	C6A—C11A	1.391 (12)
N2A—C5A	1.349 (12)	C6A—C7A	1.400 (13)
N2A—H2AA	0.8600	C6A—H6A	0.9300
N2A—H2AB	0.8600	C7A—C8A	1.378 (14)
C1A—C2A	1.394 (13)	C7A—H7A	0.9300
C1A—H1AA	0.9300	C8A—C9A	1.383 (13)
C2A—C3A	1.418 (14)	C9A—C10A	1.338 (13)
C3A—C4A	1.360 (12)	C9A—H9A	0.9300
C3A—H3AA	0.9300	C10A—C11A	1.393 (12)
C4A—C5A	1.402 (12)	C10A—H10A	0.9300
C4A—H4AA	0.9300	C11A—C12A	1.474 (13)
Br1B—C2B	1.912 (10)	O1B—C8B	1.384 (11)
N1B—C5B	1.351 (11)	O1B—H1BB	0.8200
N1B—C1B	1.359 (12)	O2B—C12B	1.312 (10)
N2B—C5B	1.318 (13)	O2B—H2BC	0.8200
N2B—H2BA	0.8600	O3B—C12B	1.250 (11)
N2B—H2BB	0.8600	C6B—C7B	1.396 (13)
C1B—C2B	1.332 (13)	C6B—C11B	1.401 (12)
C1B—H1BA	0.9300	C6B—H6B	0.9300
C2B—C3B	1.381 (14)	C7B—C8B	1.383 (14)
C3B—C4B	1.400 (12)	C7B—H7B	0.9300
C3B—H3BA	0.9300	C8B—C9B	1.379 (13)
C4B—C5B	1.410 (12)	C9B—C10B	1.358 (13)
C4B—H4BA	0.9300	C9B—H8B	0.9300
O1A—C8A	1.370 (10)	C10B—C11B	1.423 (13)
O1A—H1AB	0.8200	C10B—H10B	0.9300
O2A—C12A	1.307 (11)	C11B—C12B	1.456 (12)
			
C1A—N1A—C5A	119.3 (8)	C7A—C6A—H6A	119.7
C5A—N2A—H2AA	120.0	C8A—C7A—C6A	119.1 (8)
C5A—N2A—H2AB	120.0	C8A—C7A—H7A	120.4
H2AA—N2A—H2AB	120.0	C6A—C7A—H7A	120.4
N1A—C1A—C2A	123.1 (9)	O1A—C8A—C7A	117.9 (8)
N1A—C1A—H1AA	118.5	O1A—C8A—C9A	122.7 (9)
C2A—C1A—H1AA	118.5	C7A—C8A—C9A	119.4 (8)
C1A—C2A—C3A	118.7 (9)	C10A—C9A—C8A	121.8 (9)
C1A—C2A—Br1A	120.5 (8)	C10A—C9A—H9A	119.1
C3A—C2A—Br1A	120.8 (7)	C8A—C9A—H9A	119.1
C4A—C3A—C2A	117.0 (9)	C9A—C10A—C11A	120.7 (8)
C4A—C3A—H3AA	121.5	C9A—C10A—H10A	119.7
C2A—C3A—H3AA	121.5	C11A—C10A—H10A	119.7
C3A—C4A—C5A	122.0 (9)	C6A—C11A—C10A	118.4 (8)
C3A—C4A—H4AA	119.0	C6A—C11A—C12A	121.1 (8)
C5A—C4A—H4AA	119.0	C10A—C11A—C12A	120.5 (8)
N2A—C5A—N1A	115.5 (8)	O3A—C12A—O2A	122.8 (8)
N2A—C5A—C4A	124.6 (8)	O3A—C12A—C11A	122.3 (8)
N1A—C5A—C4A	119.9 (8)	O2A—C12A—C11A	114.9 (7)
C5B—N1B—C1B	120.1 (9)	C8B—O1B—H1BB	109.5
C5B—N2B—H2BA	120.0	C12B—O2B—H2BC	109.5
C5B—N2B—H2BB	120.0	C7B—C6B—C11B	121.1 (9)
H2BA—N2B—H2BB	120.0	C7B—C6B—H6B	119.5
C2B—C1B—N1B	121.0 (9)	C11B—C6B—H6B	119.5
C2B—C1B—H1BA	119.5	C8B—C7B—C6B	118.6 (9)
N1B—C1B—H1BA	119.5	C8B—C7B—H7B	120.7
C1B—C2B—C3B	121.9 (10)	C6B—C7B—H7B	120.7
C1B—C2B—Br1B	118.8 (8)	C9B—C8B—C7B	121.6 (8)
C3B—C2B—Br1B	119.3 (7)	C9B—C8B—O1B	118.7 (8)
C2B—C3B—C4B	117.9 (9)	C7B—C8B—O1B	119.6 (9)
C2B—C3B—H3BA	121.0	C10B—C9B—C8B	120.0 (8)
C4B—C3B—H3BA	121.0	C10B—C9B—H8B	120.0
C3B—C4B—C5B	118.7 (9)	C8B—C9B—H8B	120.0
C3B—C4B—H4BA	120.7	C9B—C10B—C11B	121.0 (9)
C5B—C4B—H4BA	120.7	C9B—C10B—H10B	119.5
N2B—C5B—N1B	117.2 (8)	C11B—C10B—H10B	119.5
N2B—C5B—C4B	122.5 (9)	C6B—C11B—C10B	117.7 (8)
N1B—C5B—C4B	120.3 (9)	C6B—C11B—C12B	119.9 (8)
C8A—O1A—H1AB	109.5	C10B—C11B—C12B	122.4 (8)
C12A—O2A—H2AC	109.5	O3B—C12B—O2B	121.2 (8)
C11A—C6A—C7A	120.6 (9)	O3B—C12B—C11B	124.0 (8)
C11A—C6A—H6A	119.7	O2B—C12B—C11B	114.8 (8)
			
C5A—N1A—C1A—C2A	2.4 (14)	C7A—C8A—C9A—C10A	−1.6 (13)
N1A—C1A—C2A—C3A	−1.6 (14)	C8A—C9A—C10A—C11A	2.3 (13)
N1A—C1A—C2A—Br1A	179.1 (7)	C7A—C6A—C11A—C10A	1.4 (13)
C1A—C2A—C3A—C4A	−0.9 (13)	C7A—C6A—C11A—C12A	−179.2 (8)
Br1A—C2A—C3A—C4A	178.4 (6)	C9A—C10A—C11A—C6A	−2.1 (13)
C2A—C3A—C4A—C5A	2.6 (13)	C9A—C10A—C11A—C12A	178.5 (8)
C1A—N1A—C5A—N2A	179.8 (8)	C6A—C11A—C12A—O3A	−178.3 (8)
C1A—N1A—C5A—C4A	−0.6 (13)	C10A—C11A—C12A—O3A	1.1 (13)
C3A—C4A—C5A—N2A	177.5 (9)	C6A—C11A—C12A—O2A	2.6 (12)
C3A—C4A—C5A—N1A	−1.9 (13)	C10A—C11A—C12A—O2A	−178.1 (8)
C5B—N1B—C1B—C2B	−1.8 (14)	C11B—C6B—C7B—C8B	−0.5 (13)
N1B—C1B—C2B—C3B	3.1 (14)	C6B—C7B—C8B—C9B	2.7 (13)
N1B—C1B—C2B—Br1B	−178.4 (7)	C6B—C7B—C8B—O1B	178.5 (7)
C1B—C2B—C3B—C4B	−2.4 (13)	C7B—C8B—C9B—C10B	−3.4 (13)
Br1B—C2B—C3B—C4B	179.1 (6)	O1B—C8B—C9B—C10B	−179.3 (8)
C2B—C3B—C4B—C5B	0.6 (12)	C8B—C9B—C10B—C11B	1.9 (13)
C1B—N1B—C5B—N2B	−178.7 (8)	C7B—C6B—C11B—C10B	−1.0 (12)
C1B—N1B—C5B—C4B	0.1 (13)	C7B—C6B—C11B—C12B	178.9 (8)
C3B—C4B—C5B—N2B	179.2 (8)	C9B—C10B—C11B—C6B	0.3 (12)
C3B—C4B—C5B—N1B	0.5 (12)	C9B—C10B—C11B—C12B	−179.5 (8)
C11A—C6A—C7A—C8A	−0.8 (13)	C6B—C11B—C12B—O3B	0.8 (12)
C6A—C7A—C8A—O1A	−177.2 (7)	C10B—C11B—C12B—O3B	−179.4 (8)
C6A—C7A—C8A—C9A	0.8 (13)	C6B—C11B—C12B—O2B	−180.0 (7)
O1A—C8A—C9A—C10A	176.3 (8)	C10B—C11B—C12B—O2B	−0.2 (11)

### Hydrogen-bond geometry (Å, °)

**Table:**

*D*—H···*A*	*D*—H	H···*A*	*D*···*A*	*D*—H···*A*
N2A—H2AA···O3A^i^	0.86	2.19	2.996 (11)	155
N2A—H2AB···O1A^ii^	0.86	2.13	2.969 (11)	166
N2B—H2BA···O3B^iii^	0.86	2.19	3.020 (11)	163
O1A—H1AB···O3B^iv^	0.82	1.87	2.688 (8)	175
O2A—H2AC···N1A^v^	0.82	1.80	2.605 (10)	168
O1B—H1BB···O3A^vi^	0.82	1.94	2.762 (8)	177
O2B—H2BC···N1B^vii^	0.82	1.85	2.663 (11)	170
C6B—H6B···O1A^viii^	0.93	2.52	3.416 (11)	161
C7B—H7B···O3A^vi^	0.93	2.58	3.262 (12)	131
C9A—H9A···O3B^iv^	0.93	2.48	3.182 (11)	132
C10A—H10A···O1B^ix^	0.93	2.53	3.416 (11)	158

Symmetry codes: (i) *x*−1, *y*−1, *z*; (ii) *x*−1/2, −*y*+1/2, *z*; (iii) *x*, *y*−1, *z*; (iv) −*x*+1, −*y*+1, *z*+1/2; (v) *x*+1, *y*+1, *z*; (vi) −*x*+3/2, *y*−3/2, *z*−1/2; (vii) *x*, *y*+1, *z*; (viii) −*x*+1, −*y*+1, *z*−1/2; (ix) −*x*+3/2, *y*+3/2, *z*+1/2.
